# Assessing biocompatibility & mechanical testing of 3D-printed PEEK versus milled PEEK

**DOI:** 10.1016/j.heliyon.2022.e12314

**Published:** 2022-12-15

**Authors:** Neil Limaye, Lorenzo Veschini, Trevor Coward

**Affiliations:** Guy’s Hospital, King's College London, London, SE1 9RT, UK

**Keywords:** Biomaterials, Polyetheretherketone, CAD-CAM, Biocompatibility, Surgery

## Abstract

**Objectives:**

To compare mechanical properties of 3D-printed and milled poly-ether-ether-ketone (PEEK) materials. To define post-production treatments to enhance biocompatibility of 3D-printed PEEK.

**Methods:**

Standardised PEEK samples were produced via milling and fused-deposition-modelling 3D-printing. To evaluate mechanical properties, tensile strength, maximum flexural strength, fracture toughness, and micro-hardness were measured.

3D printed samples were sandblasted with 50 or 125 μm aluminium oxide beads to increase biocompatibility.

Scanning electron microscopy (SEM) evaluated microstructure of 3D-printed and sandblasted samples, estimating surface roughness at scales from 1mm-1μm.

Cell adhesion on 3D printed and sandblasted materials was evaluated by culturing primary human endothelial cells and osteoblasts (HUVEC, HOBS) and evaluating cell growth over 48 h.

**Results:**

3D printed materials had lower tensile strength, flexural strength, and fracture toughness, but higher micro-hardness.

SEM analysis of 3D-printed surfaces showed sandblasting with 125 and 50 μm silica particles removed printing defects and created roughened surfaces for increased HUVEC and HOBs uniform cell adhesion and distribution. No cytotoxicity was observed over a 48h period, and all cells demonstrated >95% viability.

**Clinical significance:**

3D-printing of PEEK is an emerging technology with clear advantages over milling in maxillofacial implant production. Nonetheless, this manufacturing modality may produce 3D printed PEEK devices with lower mechanical resistance parameters compared to milled PEEK but with values compatible with natural bone. PEEK has poor osteoconductivity and ability to osseointegrate. Sandblasting is an inexpensive modality to remove irregular surface defects and create uniform micro-rough surfaces supporting cell attachment and potentially enhancing integration of PEEK implants with host tissue.

## 1 Introduction

Tissue defects resulting from neoplasm removal, trauma, or congenital disease can cause functional impairment and psychological disorder to a patient. The gold standard reconstructive treatment is autologous bone transplantation. However, autologous grafts have drawbacks such as limited supply, donor site comorbidity, and unpredictable resorption during healing. There is also no aesthetically satisfactory donor site for critical-sized, geometrically complex craniofacial defects. These challenges encouraged the development of tissue engineering by utilising alloplastic grafts to address the problems [1, 2].

Poly-ether-ether-ketone (PEEK) is a high-temperature thermoplastic polymer which demonstrates excellent chemical resistance to solvents except highly concentrated acids and is considered insoluble in biological fluids. PEEK’s implant radiolucency inside the body is beneficial as it demonstrates high resistance to electron beam and gamma radiation [3]. This means medical imaging scans such as Magnetic-Resonance-Imaging (MRI) or Computerised-Tomography (CT) do not affect its structure or create artefacts on imaging; advantageous over other biomaterials such as Titanium. PEEK elastic modulus (3–4GPa) [4] is close to that of bone and therefore PEEK is considered a viable option (other than the current gold standard titanium) for the construction of implants for large bone defect repair and dental implants [5]. Furthermore, the closer Young’s Modulus of PEEK and bone when compared to Titanium has been shown to reduce stress shielding and bone resorptive effects which may lead to implant failure [6, 7]. This was demonstrated strongly by a recent study investigating PEEK total-knee-replacement (TKR) devices versus Cobalt-chrome TKR devices in which it was found the PEEK material decreased stress shielding by 55% [8].

The material’s structural and mechanical properties are valuable with PEEK having considerable ductility, resistance to large deformative flows of compressive forces and uniaxial tension testing [9], a density of 1.32 g/cm^3^, and strong strain and fatigue resistance [10]. PEEK also has a tensile strength of 90–100 MPa [11], making its structural strength advantageous to its applications within the body. PEEK’s mechanical strength is advantageous in dental applications such as prosthodontics, orthopaedic applications such as bone replacement articulation implants, screws, nails as well as spinal fusion devices such as cages [12]. Gediminas *et al.* have demonstrated CAD-CAM (Computer-aided design-computer-aided-manufacture) milled PEEK fixed prostheses' resistance to fracture to be 2354N, a resistance which can easily manage the burden of the force of mastication around 400N [4]. PEEK has demonstrated tensile properties in similar ranges to enamel, bone and dentin, demonstrating its ability as a useful dental restorative material.

Recent advantages in additive manufacturing technologies have enabled 3D printing of PEEK thus enormously expanding the ability to manufacture patient-bespoke implants with decreased costs and material waste than current gold standard subtractive technologies such as Milling or Injection moulding [13]. Milling or machining PEEK is a subtractive process of removing material from a central workpiece to create specific shaped objects which can be driven by CAD-CAM. Nout *et al.* has shown milled PEEK implants have currently been used in cranioplasty and large skull reconstruction applications with successful outcomes [14].

3D printed patient-specific implants can have very complex geometries to reproduce defect anatomy and can also contain complex internal geometries including pores. PEEK’s dental applications thus far have included implants and frameworks for removable and fixed prostheses [5]. Other clinical applications of PEEK patient-specific implants have included cranioplasty PSIs, osteosynthesis plates, prosthetic scaphoid bone replacement and zygomatic bone replacement structures. PEEK has other applications in maxillofacial and orthopaedic surgery, including fracture fixation nails and novel joint-replacement devices [15, 16, 17]. FDM PEEK PSI devices passed all sterilisation methods in studies without any deformation of structure; emphasising the significant potential for 3D-printed PEEK structures to be utilised in clinical medicine [18]. Fused-deposition-modelling techniques (FDM) also have advantages of being cost-effective, accessible technology and less risk of material degradation.

The additive manufacturing techniques used in FDM production processes can be crucial to both the mechanical strength of PEEK devices and its ability to function as an osseointegrative scaffold when combined with materials such as hydroxyapatite. The printing parameters used in FDM techniques are important for the final characteristics of the PEEK device. Some of these parameters include temperature of nozzle and printing bed, roster angle, orientation of printing, nozzle and extrusion printing speeds as well as layer thickness [19]. These parameters can be altered to produce PEEK prints with greater mechanical strength, essential to consider in clinical applications such as spinal implants where mechanical loading is a combination of cyclic and static loads [20].

It is also important to consider double deposition of filaments in the FDM process which some studies have shown to have a detrimental effect of layer detachment and thus weakening mechanical strength [21]. Therefore, it is essential to consider altering these parameters to produce PEEK prints with greater mechanical strength.

Literature has shown FDM-produced PEEK composites surface coated with calcium hydroxyapatite (CHAp) can demonstrate strong cell attachment, boosting its viability to osseointegrate as a scaffold [22, 23]. 3D printing processes are beneficial to create porous microgrid lattices to increase bone development due to better fluid and cell body transport. Oladapo *et al.* also illustrated that FDM processes can produce 3D printed PEEK bone structures with regulated porosity with reduced weight and increased capability [24]. It has also been shown that the interconnectedness between the pores in the PEEK scaffold is essential to create a favourable environment for the development of cells and blood vessels driving nutrients and oxygen for bone growth [25]. However, it is important to note the pore size must be able to withstand mechanical stresses and maintain integrity as well as be able to be large enough to distribute nutrients and allow cell adhesion [19, 26].

Additionally, post-printing modifications to PEEK surfaces such as coating, chemical or mechanical treatment (such as sandblasting) or physical deposition methods can also be performed to increase bioactivity of PEEK implants [27]. This is by increasing the surface roughness of the material, which therefore has an increased surface area offering more binding sites for cell attachment [28]. Previous studies have investigated surface modification of PEEK material produced by traditional processes such as injection moulding and milling but there is still literature lacking on surface modification of FDM-PEEK, which this study aims to further. FDM production techniques can naturally produce peaks and valleys as the print nozzle builds the material layer-by-layer, leaving some areas unfilled between lines and layers [12]. These peaks and ridges increase surface area, allowing for more space for cells to attach and spread. Han et al. demonstrated that FDM-produced PEEK without sandblasting produced a rougher surface than one that was sandblasted and thus raises the question of why sandblast the surface post-production. This study utilised set techniques and grain sizes for sandblasting to achieve a more controlled process of producing regular and determined surface roughness in comparison to the more random surface topography that may be produced by pure FDM printing. Our sandblasting process also successfully assessed specific surface roughness on a scale of <100μm-the scale crucial to assess microtopography and differing from previous studies by addressing surface topography modulation at different scales [29, 30]. Han et al. also concluded that sandblasting can also achieve a more homogenous attachment of cells when compared to a pure-FDM produced PEEK surface [12]. To consider for biomedical applications, a post-printing process is almost always required to eliminate any printing defects that may not fit the exact precision of patient-specific implants [31]. Li et al. also found that specimens produced by FDM printing processes introduce machining errors which can also reduce mechanical strength [30]. Therefore it is crucial to balance eliminating surface impurities with the creation of a surface roughness to promote cell adhesion.

There are also limitations to post-production surface modification methods used for PEEK, including risks of contamination as well as issues with plasma treatment and surface micropatterning causing material degradation [32]. Polymer processing methods have also had issues modifying surfaces of implants of complex shapes [33].

PEEK is commonly described as biocompatible because it does not exert cell and tissue toxicity nor adverse immune reaction upon implantation. Moreover it cannot naturally integrate in the body, and it has been demonstrated that PEEK implants have an increased rate of failure due to lack of osseointegration than titanium ones [34].

Mechanically, failure to osseointegrate has been explained by poor osseoconductivity and ability of PEEK alone to support adhesion of cell involved in bone healing (osteoblasts, endothelial cells) with consequent aspecific body response (fibrotic encapsulation) upon implantation [35].

The aim of this work is to compare the mechanical properties of 3D printed versus milled PEEK samples and to improve surface topography of 3D printed PEEK samples to promote endothelial and osteoblasts cells adhesion. This study hypothesised that 3D printed PEEK would not be negatively impacted in terms of mechanical strength by FDM printing processes and that mechanical treatment using sandblasting can increase the biocompatibility of PEEK surfaces. Therefore, we aimed to produce PEEK prints using Milling and FDM printing processes with mechanical strength values close to that of natural cortical bone. The study also aimed to FDM print PEEK samples with a high printing accuracy. This was combined with specific printing parameters that were designed to produce PEEK prints with the greatest mechanical strength. Currently, there is limited available standards for FDM printing of PEEK and mechanical testing of FDM PEEK demonstrating high quality printing, and thus this study aims to add to this literature [19]. Moreover, we also aimed to alter the surface of PEEK by using mechanical treatment through sandblasting to create a roughened topography, with the goal to demonstrate increased cell adhesion and biocompatibility.

We show that although 3D printed PEEK samples had reduced mechanical strength to that of milled ones the values are still close and compatible to that of bone. Furthermore, we show that inexpensive sandblasting treatment of 3D printed PEEK surface is sufficient to abolish large irregularities on the surface and to create a micro rough topography which can support the adhesion of human endothelial cells and osteoblasts in vitro. We therefore have aimed to contribute to the literature by demonstrating sandblasting as a useful, cost-effective surface modification for PEEK which can promote the adhesion of cells and give potential for osseointegration, highlighting PEEK biocompatibility.

## 2 Materials and Methods

### 2.1 Materials

The biomaterial investigated in this project is 450G Victrex PEEK (Victrex Manufacturing Ltd, Rotherham S61 4QH) [36]. FDM samples were produced using the Apium Additive Technologies GmbH P155 printer. The Milled samples were created by milling processes using a custom modified Proxon M570 non-CNC (non-computerised control) machine.

### 2.2 3D printing models

#### 2.2.1 Fusion 360 designing of samples

**Figure 1:**
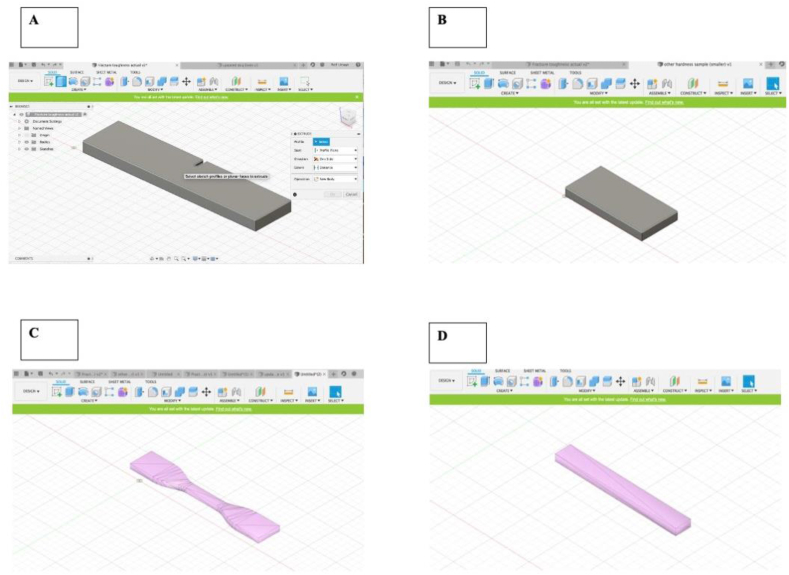
Design of PEEK specimens for mechanical testing. A) Specimen design for FT test is a notched cuboid (SENB = 6.41). B) Specimen design for VH test is a cuboid. C) Specimen design for TS is a 4mm thick dumbbell. D) Specimen for FS test is an elongated cuboid. All sample designed according to specifications in Table 1.

Fusion 360 computer-aided-design (CAD) software (Figure 1) was used to design FDM PEEK samples according to the ISO and ASTM dimensions standard (Table 1), 3D printing support structures were also included in the design. Models were exported as. *stl* files and sliced using Simplify 3D software (Simplify3D- USA, OH, 10805 Indeco Dr, Cincinnati) [37] to generate printer-ready G-code. All 3D printed samples described throughout the study were printed with the Apium P155 3D printer according to parameters listed in Tables 2 and 3 and following Apium guidelines for optimal printing and sample adhesion to building platform. After FDM-PEEK sample production, the samples’ printing supports were removed using a cutting tool evenly to leave the PEEK bodies standardised for testing.

**Table 1:** Specimen specifications for FDM-printed and Milled PEEK samples for mechanical testing.

ASTM and ISO dimensions for PEEK samples	Dimension sizes (mm)
ASTM D638 for Tensile strength	115 × 19 × 4
ASTM E384-99 for Vickers hardness	40 × 30 × 8
ASTM D5045-14 for Fracture toughness	88 × 20 × 5 (SENB = 6.41mm)
ISO 178: 2019	80 × 10 × 4

SENB = Single edged notch bend.

**Table 2:** Temperatures used in FDM printing of PEEK in this study.

Temperatures used in PEEK FDM printing processes in this study	°C
PEEK Filament loading	420
Bed calibration and Print Bed temperature (during printing)	130
Nozzle Extrusion (during printing)	485
PEEK filament roll storage temperature	60

**Table 3:** Printing parameters used for FDM PEEK printing.

Printing parameters using Apium P155 printer	
Desiccation	60 °C for 15 h
Nozzle temperature	485 °C (can be heated up to 520 °C)
Bed temperature	130 °C (can be heated up to 160 °C)
Infill Percentage (%)	100%
Machine volume (w,d,h) (mm)	590 × 620 × 680
Print bed volume (w,d,h) (mm)	155 × 155 × 155
Print volume (w,d,h) (mm)	140 × 135 × 148
Nozzle diameter	0.4mm
Number of extruders	1
Filament diameter	1.75 mm
Extrusion multiplier	0.9mm
Extrusion Width (automatic)	0.48mm
Reproducibility	0.1 mm
x/y resolution	Product resolution: 0.5 mm
Machine resolution: 0.0125 mm	
Z resolution	Product resolution: 0.1 mm
Machine resolution: 0.05 mm	
Internal & External Fill pattern	Rectilinear
Printing Speed	33.3 mm/s
Fan Speed	60%
Operating system	Mac/Windows/Linux
Slicing software used	Simplify3D
File format	STL

#### 2.2.2 Mechanical testing

Tensile Strength, fracture toughness and 3-point flexural testing was all conducted using the INSTRON 5960 Series Universal Testing System. Sample sizes were considered based on previous studies on PEEK mechanical testing [12, 30, 38, 39].

#### 2.2.3 Tensile strength

The ASTM D638 dog-bone specimens (Figure 1-A) were tested for tensile strength on the INSTRON System using a loading rate of 5 mm/s and load of 50KN. 3 Milled PEEK and 3 FDM-printed PEEK samples were produced and tested for tensile strength. The maximum tensile strength and Young’s modulus was calculated using stress-strain curves in Microsoft Excel. The raw data collected from the test included time, extension, load, tensile extension, tensile stress, tensile strain, true strain and true stress based on previous literature [40].

#### 2.2.4 Fracture toughness

The ASTM D5045-14 specimens (Figure 1-B) were tested using the INSTRON System using a loading rate of 5 mm/s and load of 50KN, with all specimen dimensions rechecked with Vernier calipers. 3 Milled PEEK and 3 FDM-printed PEEK samples were produced and tested for fracture toughness. The raw data recorded for the fracture toughness test were time, displacement, force, tensile displacement, tensile stress, and composite strain. A 1cm mark was placed for each specimen to indicate sample placement.

Similar to Tensile Strength, the data was elaborated in Excel to derive stress-strain curves and to calculate mean values. Data were Kl_c_ values were calculated according to the formula: Kic = σ√πα [41].

#### 2.2.5 Three-point-flexural testing

The ISO 178:2019 flexural bar specimens (Figure 1-C) were tested using the INSTRON System using a loading rate of 2 mm/s and load of 50KN, inputting all specimen dimensions with a support span of 49mm for each PEEK specimen. 6 Milled PEEK and 6 FDM-printed PEEK samples were produced and tested for 3-point flexural strength. The data collected was time, displacement, force, flexure stress at maximum flexure load and force at maximum flexure load. This raw data was also converted to Microsoft Excel formats and exported, mean values were calculated and put into graphical formats.

#### 2.2.6 Vickers hardness testing

The ASTM E384-99 Vickers hardness samples (Figure 1-D) were tested using a Struers Durascan 20 G5 Hardness tester. This machine was set to the Vickers indentation testing method using x50, x10 and x5 zooms with a testing load of 0.2N based on previous Vickers testing of polymers (73) and this was maintained for all testing specimens. The dwell time used in this testing method was 15 s. 6 Milled PEEK and 6 FDM-printed PEEK samples were produced and tested for Vickers hardness. Testing was performed in 3 well-illuminated regions (upper, middle, lower) randomly chosen across all samples. The following equation HV = 1.8544*P*/dˆ2 was used by the machine to calculate the Vickers Pyramid Number (HV) based on previous literature [42].

### 2.3 SEM analysis

#### 2.3.1 Sandblasting preparation

Sandblasting procedures were conducted using aluminium oxide in 50μm and 125 μm grit sizes on FDM produced. The bar pressure was set at 2 bar with the distance between the nozzle and the sample set at 3cm. After sandblasting, the residual sand grits on the samples were washed off with water under ultra-sonication for 15 min, leaving the final PEEK sandblasted samples.

#### 2.3.2 Image analysis

The characteristic and topography of the samples were investigated through SEM images. Samples were mounted on studs using conductive carbon adhesive tape and secured with conductive carbon cement. Gold coating was sputtered on the sample surface to obtain conductivity. Each PEEK sample was examined using Neoscope desktop SEM (JCM-6000 Plus, JEOL) using the following SEM observation conditions: high vacuum mode, secondary electron signal detection, 5kV accelerating voltage, standard filament current and standard probe current.

**Figure 2:**
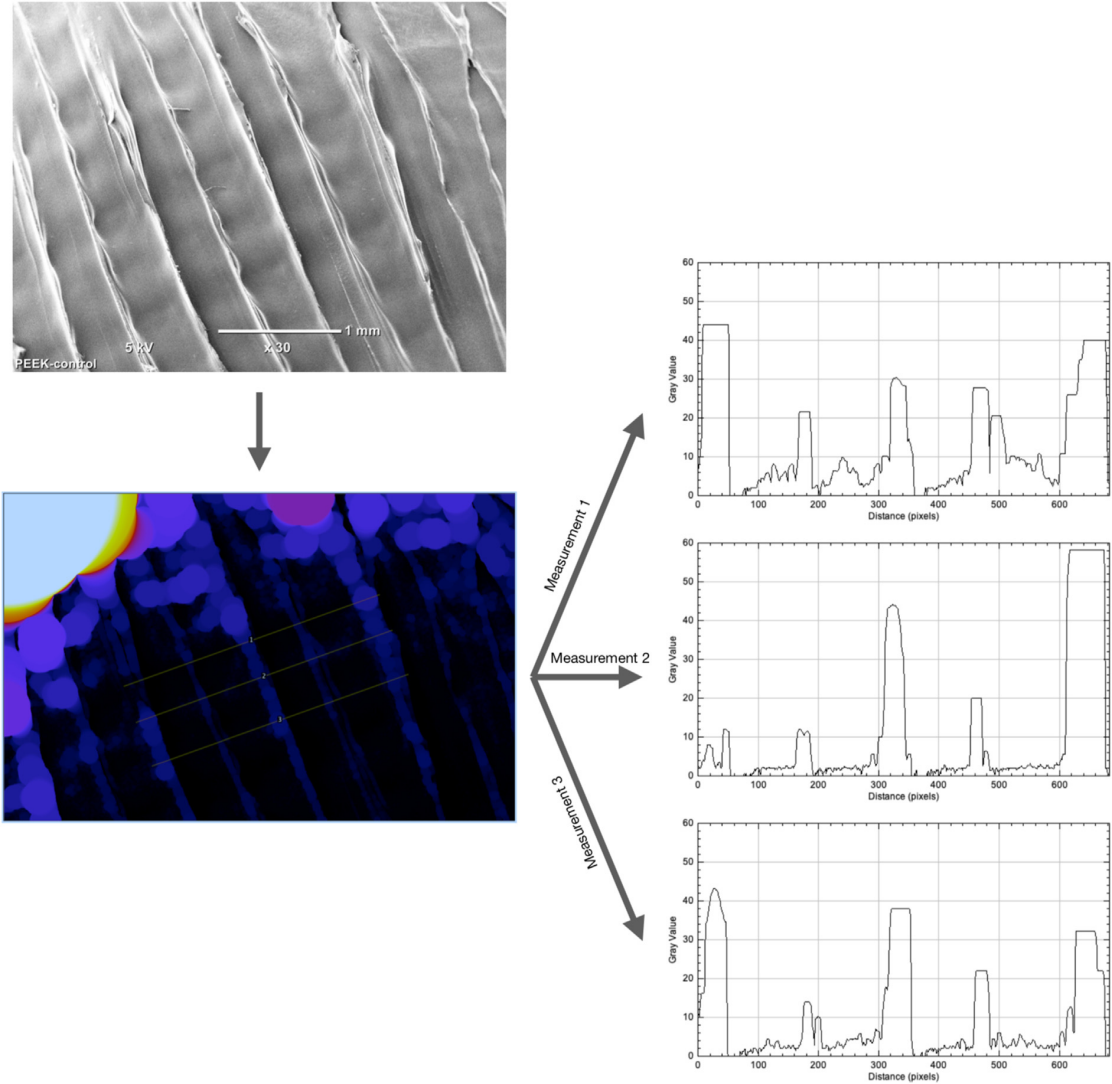
Fiji software Plot function software for image based Profilometry to measure elimination of FDM printing patterns on PEEK surface.

The elimination of printing patterns was measured by image analysis using the local thickness (complete process) computing function from Fiji software. Before assessment all SEM images were cropped to exclude the labels and scale bars. The cropped size was kept consistent for all images in the size of 1280 × 820 pixels. The local thickness computing function was then conducted. Three identical line segments crossing the printing patterns were drew on the image, and they were analysed respectively using “Plot profile” function. The “Plot profile” function generated a two-dimensional graph with the y-axis representing the intensities of pixels and x-axis representing distance along the line (Figure 2).

#### 2.3.3 Cell culture and cell adhesion experiments

To assess cell adhesion to PEEK surfaces, we seeded Human Umbilical Vein Endothelial cells (HUVEC) and Human Osteoblast cells (HOBS) obtained commercially form Promocell. Cells were sub-cultured as per provider guidelines and using recommended commercial medium (EGM2, HGM, Promocell), for adhesion experiment cells between passages 3 and 5 were used.

Cell adhesion experiments were performed by seeding 3 × 10^4^ cells (either HOVEC or HOB) per sample on either untreated or sandblasted PEEK, tissue culture plastic was used as internal positive control. Cells were allowed to adhere overnight and were then fixed with 2% paraformaldehyde (Thermo) and stained with Crystal Violet for microscopic analysis and quantification. The cells were fixated without removing non-adherent cells by washing samples twice with warm phosphate buffer saline (PBS). Cells on disks were imaged using a Heerbrugg Wild M3Z Stereomicroscope at 10X original magnification. Images were elaborated in ImageJ to measure overall area occupied by cells (blue staining) images of samples without cells were used as references for image analysis. Results are expressed as total area/field occupied by cells.

### 2.4 Statistics

Statistical analysis was performed using R studio using Student’s T test, p values < 0.05 were considered statistically significant and p values <0.01 and <0.001 highly statistically significant.

## 3 Results

### 3.1 Print accuracy

Printing accuracy is key to precise manufacturing of bespoke medical devices. We set out to evaluate the accuracy of our PEEK printer in comparison to designed dimension.

**Figure 3:**
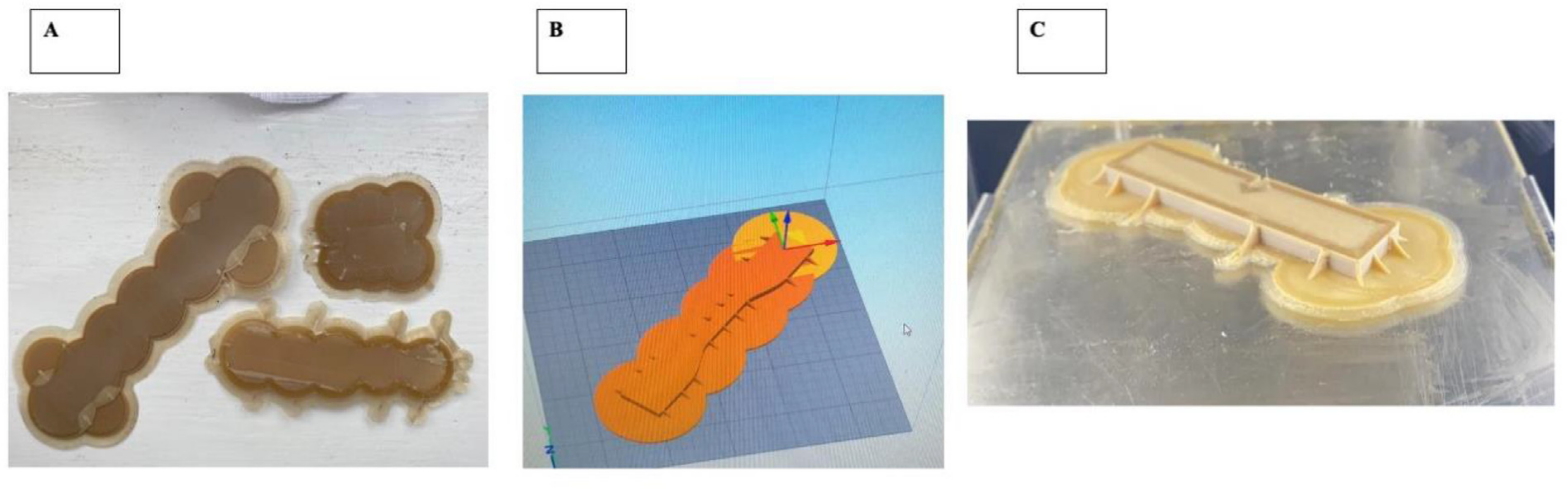
FDM Adhesion supports designed on Simplify3D software to overcome issues regarding layer-by-layer printing, stability, and infill. A) FDM printed base adhesion supports. B) PEEK tensile testing sample designed on Simplify3D. C) Successful 3D printed PEEK Fracture toughness sample with improved adhesion supports.

Our results indicate that the Apium printer consistently produced prints ∼1.8% larger than intended design therefore this discrepancy needs to be accounted when producing larger prints with high precision and dimensional accuracy (Table 4). PEEK printing is technically more challenging than conventional low temperature thermoplastics. To improve printing success rate and accuracy we added larger and increased supports by design rather than using automatically generated ones. Our results show that designed printing supports enhanced both success rate and accuracy due to better adhesion to the print bed (Figure 3, A, B, C). Print accuracy is crucial to the clinical applications of PEEK, as this biomaterial may be used in the production of complex geometrical patient-specific implants where precision is beneficial in the operation.

**Table 4:** Table showing PEEK FDM-Printing accuracy using Apium p155 printer.

	Diameter	Thickness
Disc 1	10.18	4.17
Disc 2	10.16	4.02
Disc 3	10.23	4.13
Disc 4	10.18	4.15
Disc 5	10.18	4.08
Disc 6	10.15	4.14
Disc 7	10.18	3.99
Disc 8	10.2	4.03
Disc 9	10.18	4.04
Input	10	4
Mean	10.18222222	4.08333333
Standard Deviation	0.022791324	0.06595453
Input	0.182222222	0.08333333
DFL %	1.822222222	2.08333333

### 3.2 FDM printing parameter optimisation

Moreover, the optimisation of the FDM printing parameters is crucial to produce PEEK prints with mechanical integrity to match those of normal cortical bone. Parameters such as print speed, printing bed temperature, nozzle temperature, fill patterns and infill % can be changed to FDM prints with varying porosity and mechanical strength. Table 3 demonstrates the FDM printing parameters used in this study.

### 3.3 Mechanical testing

**Figure 4:**
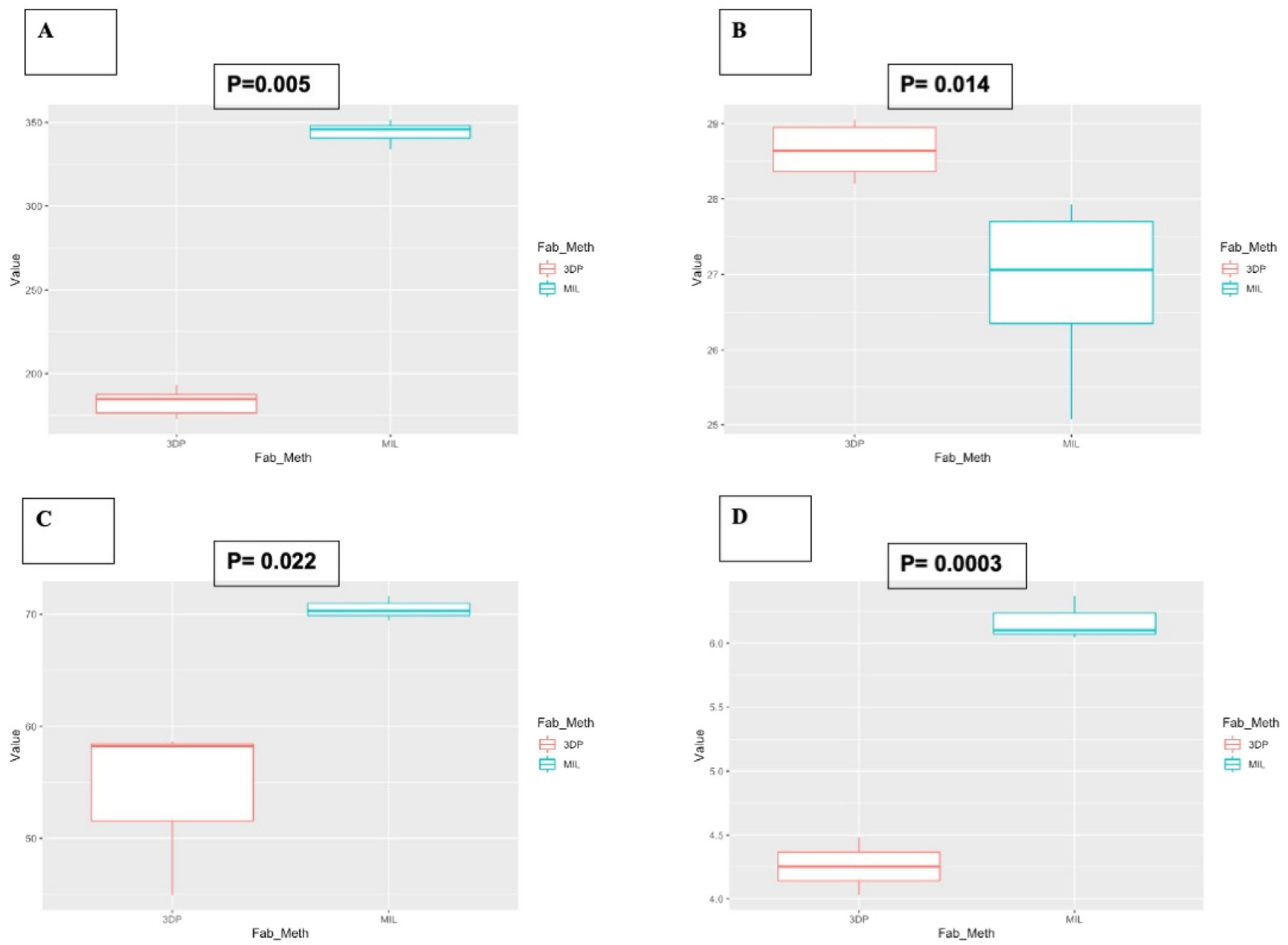
Box and plot graphs for mechanical testing. A) Box and plot graph to show statistically significant variance for flexural strength testing. B) Box and plot graph to show statistically significant variance for Vickers hardness testing. C) Box and plot graph to show statistically significant variance of tensile strength testing. D) Box and plot graph to show statistically significant variance for Fracture toughness K_IC_).

Mechanical testing demonstrated that 3D printed materials had lower tensile strength (53.91 ± 7.8 vs 70.74 ± 1.12, MPa, Figure 4-A), lower maximum flexural strength (182.78 ± 7.99 vs 344.12 ± 6.56, MPa, Figure 4-B), lower fracture toughness (4.25 ± 0.22 vs 6.17 ± 0.17, K_IC_, Figure 4-C), but higher micro hardness (29.2 ± 1.51, vs 26.85 ± 1.1, HV [N/mm^2^], Figure 4-D). 3D printed samples have significantly lower Maximum tensile strength fracture toughness and maximum flexure load. The inferior mechanical properties are compatible with that of bone supporting 3D printing as a viable technique to fabricate implantable bespoke medical devices with unmatched freedom of design. Both Milled and FDM-printed PEEK demonstrated mechanical strength values much closer to natural bone compared to other biomaterials such as Titanium, again underscoring the potential viability of this bone replacement biomaterial.

### 3.4 SEM analysis

Surface topography of implants is a well-established key factor to improve osseoinduction and osseointegration. We set to modulate surface macro and micro topography of 3D printed samples to ablate macroscopic irregularities and to enhance cell adhesion on PEEK surfaces.

**Figure 5:**
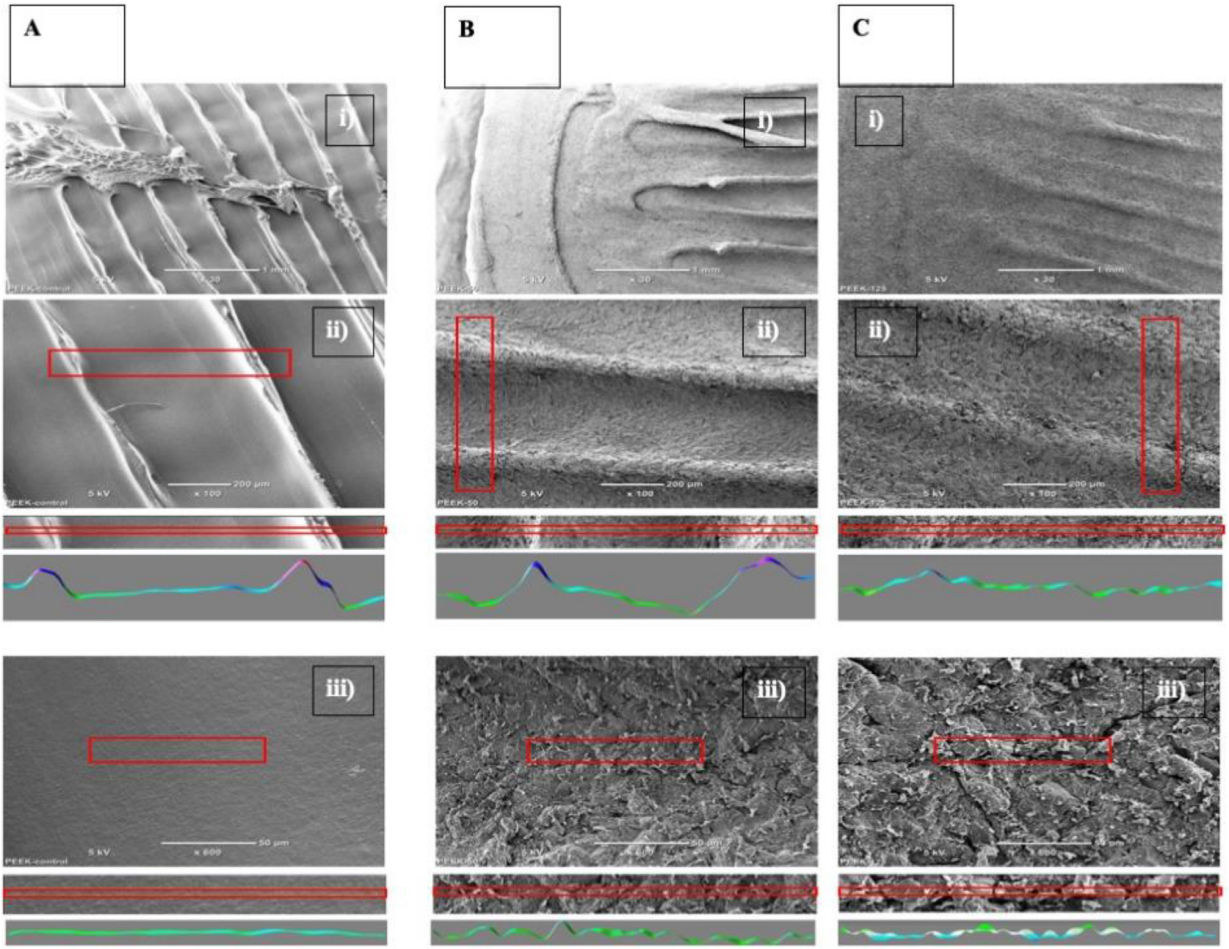
A) SEM images of untreated 3D-printed PEEK surfaces at low (A-i,30x), medium(A-ii,100x) and high (A-iii, 600x) magnification and corresponding surface analysis demonstrating large irregularities in the ∼100μm scale but otherwise smooth surface. B) SEM images of sandblasted (50μm grains) at low, medium, high magnification and corresponding image analysis demonstrating removal of surface defects and production of a roughened micro-surface. C) SEM images of sandblasted (125μm grains) at low, medium, high magnification demonstrating removal of surface defects and production of a roughened micro-surface. Image analysis plots correspond to red square on images. Scalebars 1mm, 100um and 50 um (i,ii,iii).

SEM analysis of 3D printed PEEK samples showed macroscopic irregularities created by material dragging by the printing head (Figure 5 A). High magnification (600x) of the same samples within irregularities demonstrated a very smooth surface of 3D printed PEEK.

Sandblasting with medium (50 mm, B) or large (125 mm, C) aluminium particles smoothened and abated the irregularities creating a rough micro topography less than 100 microns (Figure 5 B, C bottom panels.).

### 3.5 Cell adhesion and growth

**Figure 6:**
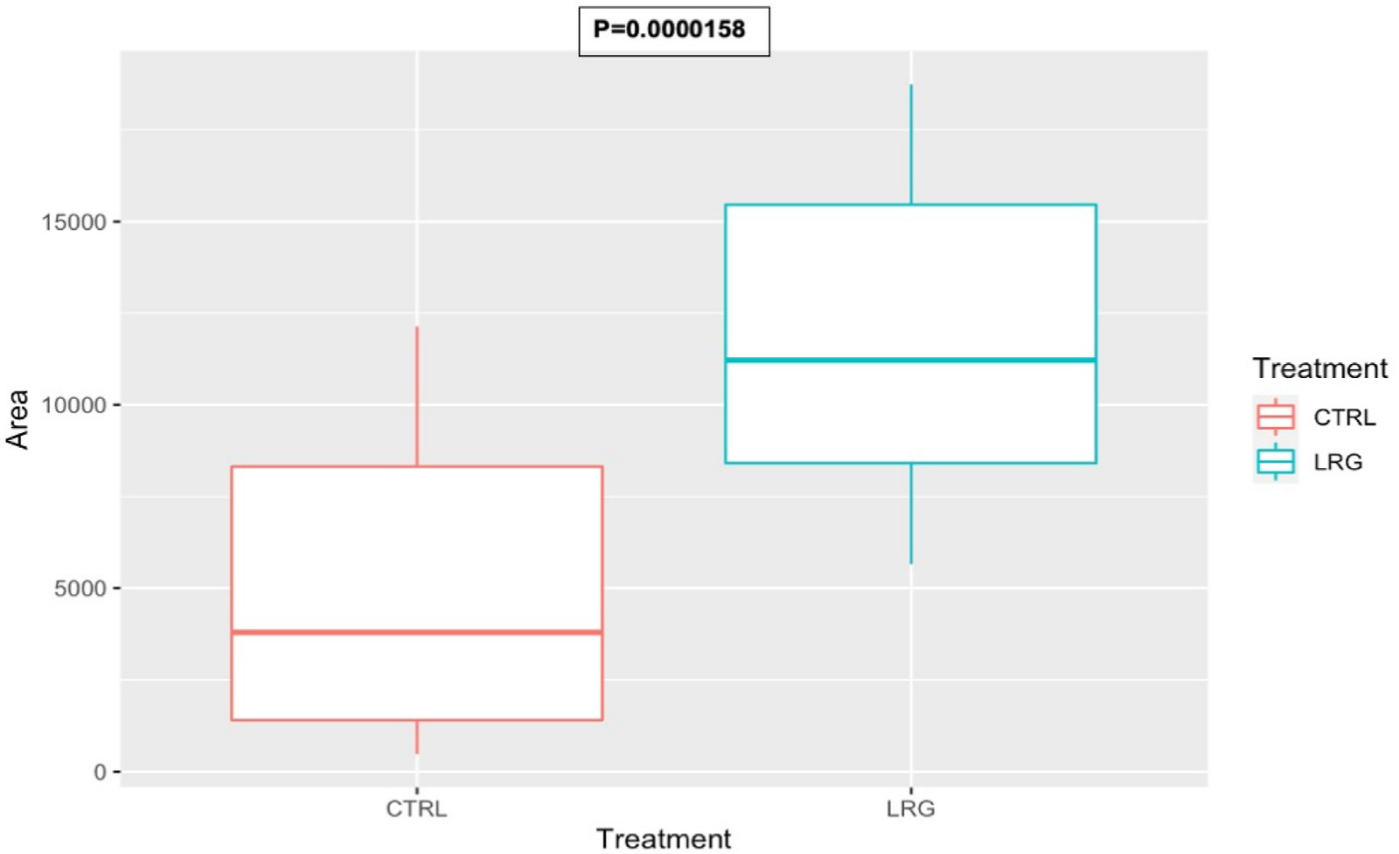
Box and plot graphs to demonstrate increased Osteoblast adhesion after sandblasting with large grains when compared to control. This is due to the creation of a roughened micro-surface with the elimination of surface defects by the sandblasting process.

**Figure 7:**
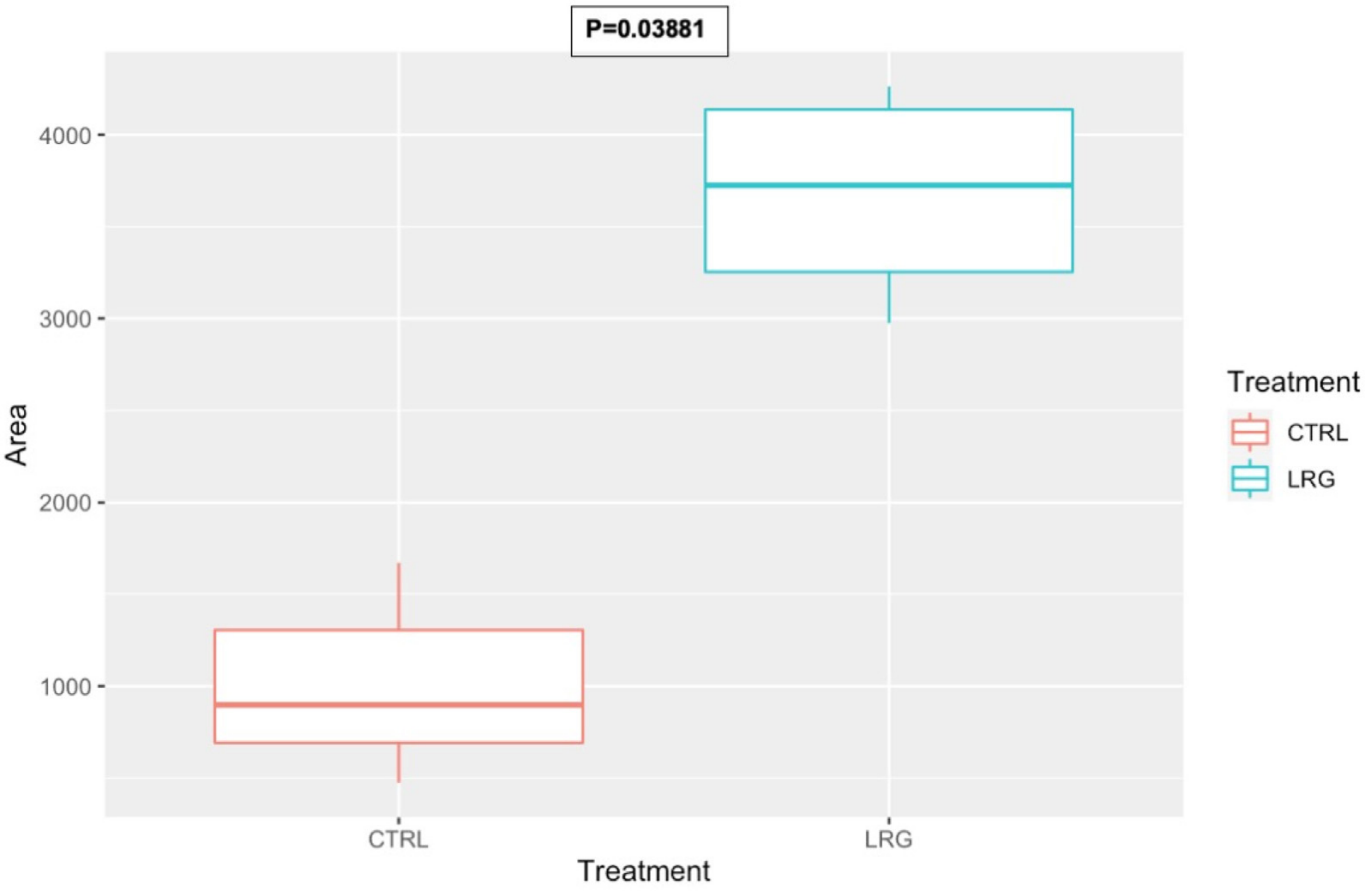
Box and plot graphs to demonstrate increased HUVEC adhesion after sandblasting with large grains when compared to control. This is due to the creation of a roughened micro-surface with the elimination of surface defects by the sandblasting process.

Cell adhesion measurements demonstrated that both HUVEC and HOB preferentially adhered to the surface of sandblasted samples while more than half the initial number of cells failed to adhere on untreated 3D printed surfaces (Figures 6 and 7) and were instead found to adhere on the bottom of the well containing the samples. Conversely, very few cells were found on the bottom of sandblasted samples or floating in culture media in all samples confirming that PEEK does not elicit direct cytotoxicity in standard culture conditions and that most seeded cells were able to adhere on the surface of sandblasted samples. The increased cell adhesion in surface treated PEEK samples is a positive finding to consider in the osseointegrative capabilities of the material when utilised as a scaffold.

## 4 Discussion

As it happened in early studies with titanium alloys in the 70’s PEEK material has clearly demonstrated potential to be employed as a versatile high-performance material for the manufacture of craniofacial implants. Modern FDM techniques have created developments in patient-specific-implants custom printed for each patient.

### 4.1 Apium P155 printing quality improvements

3D printing of PEEK is a relatively new technology and PEEK printers are becoming more accessible. In our study we used the Apium model P155 which is among the few commercially available printers certified for the production of medical devices. In comparison to printing with lower temperature thermoplastic materials such as PLA, PEEK 3D-printing poses a number of additional technical challenges such as choice of appropriate infilling strategy and the need to design bespoke 3D printing supports for maximal printing quality.

Precise bed calibration and increased and larger adhesion printing supports such as cylinders and ribs increased the accuracy of PEEK FDM printing and stabilise the layer-by-layer printing process to prevent compromise of mechanical integrity (Figure 3).

### 4.2 FDM printing parameter optimisation

Optimising FDM printing parameters for the P155 printer was crucial to the mechanical strength of 3D-printed PEEK.

The main FDM printing parameter factors that affect the mechanical properties of 3D-printed PEEK include printing speed, nozzle temperature, layer thickness, raster angle and the filling ratio [43]. In addition, other factors such as nozzle diameter, chamber temperature, scanning path, filling ratio, filament feed rate and viscosity have also shown to affect the mechanical strength of PEEK [44].

The nozzle extrusion temperature used in this study was 485 °C as recommended by the manufacturer of the printer. Various studies on FDM PEEK have been carried out with printing at extrusion temperatures of 350 °C [38], 385 °C,([45] 400–430 °C [46], and 480 °C.([47] In addition, the printing bed temperature (130 °C) used in this study was also based on ideal print bed temperatures observed in a number of other studies [43].

3D printing speed of PEEK specimens has been observed as a factor affecting the mechanical strength of the material. The printing speed in this project was 33.3 mm/s (default pre-set). However, other studies have used 40, 50 and 60 mm/s. Wang *et al.* concluded that the ideal printing speed to optimise this parameter for mechanical properties should be in the range of 40–80 mm/s [43]; and therefore, for future work using this printer it may be better to utilise a print speed in this range. Another study suggested that slower printing speeds can increase porosity of PEEK models produced, reducing mechanical strength [48]. This can be a clinical problem, especially in PEEK weight-bearing applications where functions of protection and stability are crucial. Although slower print speeds may reduce mechanical strength this study has shown to find values for FDM PEEK in the ranges of other studies mentioned above.

### 4.3 Mechanical testing of PEEK

Both FDM PEEK and Milled PEEK were tested and evaluated for tensile strength, 3-point flexural strength, fracture toughness and Vickers hardness.

As Tables 5, 6, and 7 demonstrate, milled or injection moulded PEEK shows higher maximal tensile strength, flexural strength and fracture toughness compared to FDM-printed PEEK. Wang *et al.* have suggested that this difference is due to injection moulding introducing external pressures which reduces internal defects and improves the density of parts [49]. Other studies have suggested milled specimens have a more homogenous structure, with FDM PEEK being more heterogenous structure because of the presence of weld lines between deposed threads, as different orientations of these lines can lead to reduced mechanical properties [50, 51]. Our study found the maximum tensile strength of the FDM PEEK was 76.5% of the maximum tensile strength of milled PEEK, thus in line with previous literature.

**Table 5:** Table comparing Tensile strength of FDM-printed PEEK, Milled PEEK and Natural cortical bone.

Study	Year	FDM PEEK (MPa)	Milled PEEK (MPa)	Natural cortical bone (MPa)
This study	2021	53.91 (mean)	70.47	
VICTREX ref value	2020	98.00		
Wu et al. [38]	2016	56.60		
Deng et al. [82]	2018	40.00		
Rahman et al. [83]	2016	74.49		
Vaezi et al. [46]	2015	75.06		
Berretta et al. [84]	2017	90.00		
Cicala et al. [85]	2018	69.04		
Han et al. [12]	2019	95.21		
Li et al. [86]	2019	146.00		
Shanmugam et al. [87]	2021	48–265		
Arif et al. [88]	2018	82.58		
Muhsin et al. [11]	2019		118.00	
Zong et al. [39]	2019		96.40	
Oladapo et al. [19]				29.6
Havaldar et al. [89]	2014			39.74
White et al. [90]	2007			50.00–150.00
Dones et al. [91]	2010			101.9

**Table 6:** Table comparing Fracture toughness of FDM-printed PEEK, Milled PEEK and Natural cortical bone.

Study	Year	FDM PEEK (MPa·m1/2)	Milled PEEK (MPa·m1/2)	Natural cortical bone (MPa·m1/2)
This study	2021	4.25	6.17	
Sobieraj et al. [92]	2009		5.16	
Gensler et al. [41]	1996		6.30	
Friedrich et al. [93]	1989		7.20	
White et al. [90]	2007			12.00
Norman et al. [94]	1995			4.32
Wang et al. [95]	2002			5.10

**Table 7:** Table comparing Flexural strength of FDM-printed PEEK, Milled PEEK and Natural cortical bone.

Study	Year	FDM PEEK (MPa)	Milled PEEK (MPa)	Natural cortical bone (MPa)
This study	2021	182.79	344.13	
Victrex reference	2020	165.00		
Schwitalla et al. [96]	2015		318.21	
Vaezi et al. [46]	2015	132.37		
Rahman et al. [83]	2016	111.70		
Wu et al. [38]	2016	56.10		
Han et al. [12]	2019	140.83		
Li et al. [86]	2019	146.00	130.00	
Hu et al. [45]	2019	120.20		
Arif et al. [88]	2018	142.00		
Zong et al. [39]	2019		147.66	
Jeng et al. (SCFR-PEEK) [97]	2000		375.70	
Keller at al. [58]	1990			238.20
Hubbard et al. [98]	1971			82.00
White et al. [90]				50.00–150.00

By observing the path of the crack propagation using numerical simulation specific FDM processes can increase the fracture toughness of PEEK. This is achieved by depositing filament along the same trajectories that mechanical stresses are applied to; providing the material with extra reinforcement [51]. FDM processes were proven to increase the maximal tensile load the specimens could withstand, and this is clinically beneficial as PEEK is used in weight bearing and stress resistant applications.

Table 8 demonstrates that FDM PEEK showed higher Vickers hardness compared to milled PEEK, an aspect only few current studies investigating. It has been theorised that the higher value in FDM printed PEEK is due to good bonding between printing layers due to horizontal orientation when printing; as the FDM method causes layers to be ordered on the same direction, giving the printed specimen a ‘brick-like’ structure [51]. As Milled samples do not have this ordered layering, it can lead to reduced resistance against the indenter, thus giving the lower HV value.

**Table 8:** Table comparing Vickers hardness of FDM-printed PEEK, Milled PEEK and Natural cortical bone.

Study	Year	FDM PEEK (HV)	Milled PEEK (HV)	Natural cortical bone (HV)
This study	2021	29.3	26.9	
Liu et al. [99]			21.3	
Kassem et al. [100]	2019		28.2–37.5	
Kumar et al. [101]	2018		43.0	
Wei at al. [102]	2019		26.9	
Yin et al. [103]	2019			38.52

Both FDM PEEK and milled PEEK demonstrate tensile strength, flexural strength, fracture toughness and Vickers hardness values close to those of natural cortical bone, supporting PEEK’s applications as a bone replacement material. This includes applications in clinical dentistry, including different types of crowns, maxillofacial prosthesis, fixed partial dentures and removable partial dentures [52]. PEEK can be utilised in patient-specific reconstructive facial surgery within interlocking systems in zygomatic and jaw deformities [16, 53]. PEEK’s strong fracture toughness data seen in this study supports its ability as a dental material containing a crack to resist fracture, important in masticatory processes such as biting into hard food materials [54]. PEEK has also shown to have better fracture toughness properties than other dental materials such as ceramics-e.g. Zirconia [52, 55]. This investigation also demonstrated PEEK Young’s modulus values close to those of natural cortical bone, (milled = 4.12GPa, FDM = 1.80GPa, cortical bone 9.82–20.7GPa) [56, 57, 58]. The Young’s modulus of PEEK can also specially be adjusted by changing orientations of fibres and their lengths to even closely match the YM of cortical bone [59]. PEEK’s closer Young’s Modulus to that of bone in comparison to other bone replacement materials such as Titanium (which can have YM of up to 120 GPa) [60] is beneficial due to less stress shielding and less eventual osteoporotic damage. PEEK’s elastic modulus can be further increased by reinforcement with other materials such as carbon-fibre or glass-fibre to produce PEEK composite materials, and studies have shown higher YM values of PEEK composites such as CFR-PEEK (18 GPa) [61] and GFR- PEEK (12 GPa) [62]. The Vickers hardness value demonstrated in this study for FDM PEEK was closer to the HV of natural bone, a good indicator of its viability and better in comparison to implants with much higher HV values such as Titanium which may cause bone penetration and implant failure [63]. PEEK’s useful hardness has also been shown to be a recommending factor as a framework material for fixed dental prostheses [64, 65]. Vickers hardness is an important factor for PEEK’s application in fixed dental prostheses to manage the forces and pressures of mastication and prevent the formation of micro crack propagation. Previous literature has also identified Vickers hardness as an important mechanical factor in influencing the longevity of biomaterials within the oral cavity [66].The microstructure mechanical integrity is also a crucial aspect to investigate in dental prostheses or Orthopaedic applications as complex load patterns are applied [67].

### 4.4 Sandblasting, topography, and cell adhesion

Han *et al.* pointed out the importance of eliminating the printing patterns as cells tend to reside in the deep valley of the printing patterns and lead to a heterogeneous cell distribution [12]. The elimination of printing patterns using methods such as sandblasting can help achieve a uniform cell attachment. In the present study, the raw SEM images and further analysis with ImageJ confirmed that sandblasting is a viable method for eliminating printing patterns. The results from this study demonstrate that sandblasting is a useful and effective method to promote homogeneous cell adhesion on PEEK surfaces (Figures 6 and 7).

An evenly distributed surface roughness was also created through sandblasting. Sandblasting has demonstrated the ability to produce an enhanced surface roughness which promotes osteoconduction, bone to implant contact and increased removal torque; thus supporting sandblasted PEEK’s viability as a bone implant [68].

Previous studies confirmed the cytocompatibility of sandblasted PEEK [12, 69]. The sandblasting treatment performed well on eliminating printing patterns and achieving a homogeneously roughened surface, which would help achieve a uniform cell attachment. As Figures 6 and 7 demonstrate, post-sandblasted PEEK showed increased HUVEC and HOBS cell adhesion due to the creation of a uniform roughened micro-surface. The results from this study show that sandblasting of PEEK surfaces not only removes 3D printing artifacts at the microscopic scale but also create a more favourable environment for cell adhesion. Since no additional coating or functionalisation was performed on samples, our experiment also suggests that physical alteration of PEEK surface roughness might be sufficient to improve material’s cytocompatibility. Sandblasting has shown to be a cost-effective method of increasing bioactivity of PEEK surface, beneficial for its sustainable applications in bone integration. Previous literature has also demonstrated creating a surface roughness such as this to increase bioactivity, cell metabolic activity, proliferation and increased osteoblast adhesion [70, 71]. This suggests that sandblasting is an effective method of increasing cell adhesion and implant osseointegration compared to polished or untreated PEEK surfaces. Cell adhesion is a crucial aspect of osseointegration which anchors implants and reduces risk of implant failure. Osseointegration of PEEK devices under cyclical loading in animal models is important to comprehend the viability of PEEK as a bone replacement biomaterial. Although this in-vitro study demonstrated strong potential for PEEK, other studies have completed in-vivo investigations to further this. El Awadly *et al.* demonstrated that sandblasted ceramic filled PEEK dental implants were able to osseointegrate in 9 experimental dogs; concluding that the aluminium oxide particle blasting created a PEEK surface topography which can promote the migration, distribution, and proliferation of osteoblasts to the surface-a crucial aspect to osseointegration. The study suggested this can create contact osteogenesis which could give potential to continuous bone remodelling under cyclical loading, with bone density statistics comparable to those of Titanium [72]. Moreover, another study which investigated implanted PEEK femoral implants also demonstrated good osseointegration, limited inflammatory response and strong bone remodelling under load. The study also underscored that modification of the PEEK surface increased bone implant contact compared to untreated surfaces; supporting sandblasting to promote cell adhesion to increase bone growth [73]. Other studies have also shown PEEK strong biocompatibility in vivo and surface modifications can benefit the surface to achieve osseointegration by promoting cell adhesion [74, 75]. This investigations adds to the previous body of knowledge by adding a proof of concept using an inexpensive method of treating 3D printed PEEK to increase biocompatibility, giving the potential for 3D printed PEEK to be utilised in in vivo models such as the injection moulded PEEK studies mentioned above.

As seen in the SEM analysis (Figure 5), the FDM process of production produces grooves in the 3D-printed PEEK samples. Han *et al.* showed grooves and increased surface roughness have shown to be factors that support the adhesion and accumulation of cells, further emphasising the benefits of sandblasting and FDM production [70].

## 5 Conclusions

In this current study it can be concluded that the data obtained revealed that 3D-printing of PEEK generated objects with substantially lower mechanical properties (tensile, flexural, fracture toughness) compared to Milled PEEK. However, FDM PEEK mechanical properties remain in a range very similar to natural cortical bone, and thus both types of PEEK can be considered a valid replacement to other bone substitute biomaterials such as Titanium. The closer mechanical strength values of both types of PEEK with bone increases the viability of PEEK’s clinical applications with potential for less stress shielding effects, better osseointegration as a scaffold, and less chance of implant failure. The current lack of significant studies evaluating the mechanical properties of FDM PEEK and PEEK produced by Milling processes is an aspect that this research adds value too and has furthered.

FDM PEEK was successfully 3D printed with promising results in accuracy, diameter, and thickness, increasing the potential of FDM printing for designing complex patient-bespoke objects with increased efficiency and less waste of material. The high print accuracy demonstrated in this study is an important aspect when considering the potential of complex geometric PEEK implants. It is also beneficial to consider the outcomes of utilising different FDM printing parameters when printing PEEK devices, and how to apply these to different clinical applications when balancing mechanical strength and interconnected porosity.

The SEM image confirmed the effect of sandblasting treatment on eliminating the printing patterns and creating homogenous roughness, thus proving us a useful, cost-effective method of increasing PEEK biocompatibility. This study also signified the advantages of sandblasting as a form of mechanical treatment enhancing surface topography, as the results showed increased osteoblast and HUVEC cell adhesion with treated surfaces when compared to untreated. This can again give a potential cost-effective method of enhancing PEEK surface biocompatibility which may give clues to potential osseointegrative capabilities for PEEK scaffolds.

It is worth considering the limitations of this study with guide to future work. Furthermore, future studies may consider adding FTIR or X-ray diffraction analyses to the 3D-printed and Milled PEEK specimens to understand the differences in crystallinity in the two production methods and how this can affect the mechanical strength of the material [76, 77, 78] PEEK with increased crystallinity may show better mechanical properties and so may be more beneficial as an implant material that can weight-bear high loads or stresses [79, 80].

Despite a small sample size, we have standardised in-vitro experimental conditions to minimise this issue. The relatively small standard deviation observed in most groups seems to confirm this and thus we have assumed that the data are likely normally distributed as typical in in-vitro setups [81].

Further detailed examination of surface chemical compositions will be necessary to ensure no contamination was caused by the surface treatment. The successful cell adhesion and zero toxicity suggests there were no toxic contaminants present. However, it may also be beneficial in future studies to undertake more comprehensive industrial cleaning processes post-surface treatment and consequent biocompatibility assessment to ensure the absence of surface contaminants.

Finally, in vivo studies under physiological load using 3D printed PEEK will be necessary to evaluate osseintegration and long-term success of implants.

In conclusion, PEEK has many current applications in dentistry and maxillofacial reconstructive surgery and 3D printing significantly expands the repertoire of possible designs with great promises for the manufacture of bespoke medical devices and implants.

## Declarations

### Author contribution statement

Dr Neil Limaye: Conceived and designed the experiments; Performed the experiments; Analyzed and interpreted the data; Wrote the paper.

Dr Lorenzo Veschini: Conceived and designed the experiments; Analyzed and interpreted the data; Wrote the paper.

Professor Trevor Coward: Conceived and designed the experiments; Contributed reagents, materials, analysis tools or data; Wrote the paper.

### Funding statement

This research did not receive any specific grant from funding agencies in the public, commercial, or not-for-profit sectors.

### Data availability statement

Data included in article/supp. material/referenced in article.

### Declaration of interest’s statement

The authors declare no conflict of interest.

### Additional information

No additional information is available for this paper.
